# Postoperative Nausea and Vomiting in Female Patients Undergoing Laparoscopic Gastrointestinal Surgery with Double Prophylactic Therapy

**DOI:** 10.1055/s-0044-1787305

**Published:** 2024-06-03

**Authors:** Chunmeng Lin, Jing Li, Qian Wu, Tongfeng Luo, Zhinan Zheng

**Affiliations:** 1 Department of Anesthesia, The Sixth Affiliated Hospital, Sun Yat-sen University, Guangzhou, China; 2 Biomedical Innovation Center, The Sixth Affiliated Hospital, Sun Yat-sen University, Guangzhou, China; 3 Center for Surgery and Anesthesia, The Sixth Affiliated Hospital, Sun Yat-sen University, Guangzhou, China; 4 Center for Clinical Research, The Sixth Affiliated Hospital, Sun Yat-sen University, Guangzhou, China

**Keywords:** postoperative nausea and vomiting, female patients, laparoscopic gastrointestinal surgery, risk factor

## Abstract

**Purpose** Postoperative nausea and vomiting (PONV) is a major problem after surgery. This study aimed to demonstrate the incidence of PONV and the potential associated factors in female patients undergoing laparoscopic gastrointestinal surgery against the background of double prophylactic therapy.

**Methods** Our retrospective study recruited 109 female patients undergoing laparoscopic gastrointestinal surgery with double prophylactic therapy, combining palonosetron with dexamethasone, from October 2020 to March 2021, at the Sixth Affiliated Hospital of Sun Yat-sen University, Guangzhou, China. Patient characteristics and perioperative management factors were included in univariate and multivariate analyses to identify factors influencing PONV.

**Results** Four patients lacked complete records, and of the 105 patients included in the final analysis, 53 (50.5%) patients developed PONV. Two influencing factors for PONV were identified: a history of chemotherapy (odds ratio [OR] 0.325, 95% confidence interval [CI] 0.123–0.856; *p* = 0.023) and dosage of hydromorphone ≥ 0.02 mg/kg (OR 2.857, 95% CI 1.247–6.550; *p* = 0.013). The performance of the multivariate logistic regression was evaluated by analyzing receiver operating characteristic curves, resulting in an area under the curve value of 0.673.

**Conclusion** The incidence of PONV remains high in female patients undergoing laparoscopic gastrointestinal surgery, even with double prophylactic therapy. A dosage of hydromorphone ≥ 0.02 mg/kg may increase risk of PONV, whereas a history of chemotherapy might be a protective factor.

## Introduction

Postoperative nausea and vomiting (PONV) is a major problem after surgery in clinical nursing, occurring in 30% of the general surgical population and can be as high as 60 to 80% in high-risk populations, without prophylactic therapy. 1 2 The incidence of PONV varies among patients with different characteristics. Female sex, a history of PONV and/or motion sickness, nonsmoking status, and use of postoperative opioids are considered to be risk factors. 2 Furthermore, certain types of surgery, such as laparoscopic surgery, may be associated with an increased risk of PONV. 3 4 Gastrointestinal surgery also promotes PONV due to the handling or rotating of the stomach or bowel. 5 6 Therefore, PONV may be particularly common in high-risk patients undergoing laparoscopic gastrointestinal surgery; however, incidence is currently unclear.

PONV is associated with significant patient distress and adverse outcomes, such as electrolyte disorder, acid-base imbalance, delayed recovery, aspiration, esophageal dehiscence, or suture dehiscence. 7 8 Severe vomiting can be disastrous for gastrointestinal anastomoses. Therefore, better control of PONV is particularly important in gastrointestinal surgery.

Antinausea and antivomiting medications, such as serotonin antagonists (e.g., ondansetron), dopamine antagonists (e.g., droperidol), and corticosteroids (e.g., dexamethasone), are commonly used to prevent and treat PONV. These medications work by targeting different receptors and neurotransmitters involved in the emetic pathway, effectively reducing the occurrence and severity of PONV. The current guidelines recommend combination antiemetic therapy. 9 The rationale behind combination therapy is that different medications target different receptors and neurotransmitters involved in the emetic pathway, maximizing the antiemetic effect. By utilizing medications with complementary mechanisms of action, the risk of PONV can be further reduced. The combination of serotonin antagonists with dexamethasone is a common dual therapy used to prevent PONV for high-risk patients undergoing various surgical procedures, such as laparoscopic bariatric surgery (palonosetron 0.25 mg plus dexamethasone 10 mg), laparoscopic cholecystectomy (palonosetron 0.075 mg plus dexamethasone 8 mg), and cesarean delivery (palonosetron 0.075 mg plus dexamethasone 4 mg). 10 11 12 13 14 Even with double prophylaxis, PONV may still occur frequently. 5 15 Searching for factors related to PONV in various clinical situations may contribute to better PONV control. Thus, PONV should be investigated in specific populations and surgery types. 16

Therefore, we designed this retrospective study to elucidate the incidence of PONV and the potential factors associated with PONV in female patients undergoing laparoscopic gastrointestinal surgery against the background of double prophylactic therapy comprising palonosetron and dexamethasone.

## Materials and Methods

### Study Design and Population

This was a single-center retrospective observational study. Institutional review board exemption (approval no. 2023ZSLYEC-144) was obtained from the Sixth Affiliated Hospital of Sun Yat-sen University, Guangzhou, China. The need to obtain written informed consent from participants was waived because no treatment interventions were provided, and protected health information was not collected or analyzed. This study was conducted in accordance with the Strengthening the Reporting of Observational Studies in Epidemiology guidelines.

The inclusion criteria were female patients aged 18 to 75 years, who underwent laparoscopic gastrointestinal surgery with total intravenous anesthesia, and who received a combination of dexamethasone (5 mg, Tianjin KingYork Group Hubei TianYao Pharmaceutical Co., Ltd, Hubei, China) and palonosetron (0.25 mg, Qilu Pharmaceutical Co., Ltd, Hainan, China) at induction for double prophylaxis for PONV, and postoperative analgesia with hydromorphone (Humanwell Healthcare Co., Ltd, Hubei, China) via pump. The exclusion criteria were preoperative use of medications with known antiemetic properties and patients with incomplete data.

Many factors associated with PONV reported by previous studies, such as sex, type of surgery, type of anesthesia, prophylactic therapy for PONV, and postoperative analgesic regime, were fixed in the relatively strict inclusion criteria to control bias as much as possible.

### Data Collection

Data were collected retrospectively from October 2020 to March 2021. All data were obtained from electronic medical records. We retrieved all the demographic and clinical data of all subjects in this study, including age, sex, medical history (hypertension, diabetes mellitus, coronary heart disease, chemotherapy, radiotherapy, smoking, motion sickness, or history of PONV), laboratory values (hemoglobin and albumin levels), duration of surgery, type of surgery, fluid balance, and types and dosage of anesthetic drugs. All data were entered in a timely manner in the electronic medical record system during hospitalization and were not recalled by telephone, to minimize recall bias.

### Outcomes

The outcome was PONV during the first 24 hours after surgery. PONV can be classified into four grades: (1) grade I: no nausea or vomiting reported; (2) grade II: only nausea, but no vomiting; (3) grade III: significant vomiting without the presence of gastric content; and (4) grade IV: severe vomiting with the presence of gastric content. 17 Nausea was defined as a feeling of the urge to vomit. Retching was defined as an unproductive attempt to vomit stomach contents. Vomiting was defined as episodes of expulsion of the gastric content. According to our clinical routine, anesthetists followed up patients once a day after surgery to investigate the status of PONV and pain, and recorded this timely on the sheet of postoperative analgesic follow-up. When this study started, information on PONV was collected from previous follow-up records or from nursing records in the ward.

### Statistical Analysis

All eligible patients hospitalized from October 2020 to March 2021 were included. All continuous, normally distributed variables were summarized using mean and standard deviation. All continuous, nonnormally distributed variables were summarized using median and interquartile range. All categorical variables were summarized using frequencies and percentages. Differences were investigated as follows: Student's *t* -test for normally distributed continuous variables, Mann–Whitney *U* test for nonnormally distributed continuous variables, and Pearson's chi-squared test or Fisher's exact test for categorical variables. A univariate logistic regression analysis was performed to evaluate associations of variables with PONV, describing the odds ratio (OR) with their respective 95% confidence interval (CI). Univariate analysis was initially used to identify variables that could potentially serve as predictors of PONV. Variables with a *p* -value less than 0.1 in the univariate analysis were then introduced into a multivariate logistic regression model using the forward method to determine the final associated factors. The performance of the multivariate logistic regression model was assessed using receiver operating characteristic (ROC) curves, and the area under the curve (AUC) was calculated. Statistical analyses were performed using SPSS v22.0 (IBM SPSS Inc., Armonk, NY). All *p* -values were two-sided, and statistical significance was set at *p* < 0.05.

## Results

Among 109 patients who met the eligibility criteria, 4 patients lacked complete records. Thus, 105 patients were included in the final analysis. During the first 24 hours after surgery, 53 patients (50.5%) experienced PONV (case group), while 52 patients (49.5%) did not (control group). Demographic and clinical characteristic features in both groups are shown in Table 1 . Among the case group, the incidence of nausea was 11.4% and the incidence of vomiting was 39.1%. The incidence of PONV for grade II, III, and IV were 11.4, 17.2, and 21.9% in the case group, respectively.

**Table 1:** Demographic characteristics of patients with and without PONV analyzed by univariate logistic regression

	Case group ( *n* = 53)	Control group ( *n* = 52)	Univariate OR [95% CI]	*p* -Value
Age > 55 y	29 (54.7)	33 (63.5)	0.696 [0.318–1.520]	0.363
Body mass index ≥ 24 kg/m ^2^	14 (26.4)	19 (36.5)	0.623 [0.271–1.432]	0.256
ASA			0.588 [0.133–2.597]	0.483
1	5 (9.4)	3 (5.8)		
2	48 (90.6)	49 (94.2)		
History of PONV and/or motion sickness	31 (58.5)	22 (42.3)	1.921 [0.885–4.172]	0.099
No-smoking status	50 (94.3)	50 (96.2)	0.667 [0.107–4.163]	0.664
History of radiotherapy before surgery	4 (7.5)	5 (9.6)	1.303 [0.330–5.151]	0.706
History of chemotherapy before surgery	8 (15.1)	19 (36.5)	0.309 [0.121–0.791]	0.014
History of hypertension	6 (11.3)	9 (17.3)	0.610 [0.200–1.856]	0.384
History of diabetes mellitus	5 (9.4)	4 (7.7)	1.250 [0.316–4.940]	0.750
Preoperative albumin level (g/L) < 35 g/L	8 (15.1)	12 (23.1)	0.593 [0.220–1.596]	0.301
Preoperative hemoglobin level (g/L) < 90 g/L	13 (24.5)	8 (15.4)	1.787 [0.671–4.759]	0.245
Types of surgery			0.984 [0.536–1.806]	0.958
Gastrectomy resection	3 (5.7)	6 (11.5)		
Colon resection	29 (54.7)	22 (42.3)		
Rectum resection	21 (39.6)	24 (46.2)		
Duration of general anesthesia ≥ 180 min	42 (79.2)	38 (73.1)	1.407 [0.570–3.472]	0.459
Duration of surgery ≥ 180 min	35 (66.0)	32 (61.5)	1.215 [0.548–2.697]	0.632
Fluid balance ≥ 9 mL/kg/h	30 (56.6)	28 (53.8)	1.118 [0.518–2.414]	0.776
Dosage of propofol ≥ 7 mg/kg/h	22 (41.5)	25 (48.1)	0.766 [0.355–1.657]	0.499
Dosage of remifentanil ≥ 0.15 ug/kg/min	31 (58.5)	31 (59.6)	0.955 [0.438–2.078]	0.907
Dosage of hydromorphone ≥ 0.02 mg/kg	29 (54.7)	15 (28.8)	2.981 [1.329–6.685]	0.008
Dosage of neostigmine ≥ 0.017 mg/kg	25 (47.2)	19 (36.5)	1.551 [0.710–3.385]	0.271
Installation of stomach tube	9 (17.0)	7 (13.5)	1.315 [0.450–3.840]	0.617

Abbreviations: ASA, American Society of Anesthesiologists; CI, confidence interval; OR, odds ratio; PONV, postoperative nausea and vomiting.

Note: Data are presented as *n* (%).

Based on the results of the univariate logistic regression analysis in Table 1 , three variables demonstrated statistically significant associations with PONV ( *p* < 0.1). These included: history of PONV and/or motion sickness ( *p* = 0.097), history of chemotherapy before surgery ( *p* = 0.014), and dosage of hydromorphone ( *p* = 0.005).

**Fig. 1:**
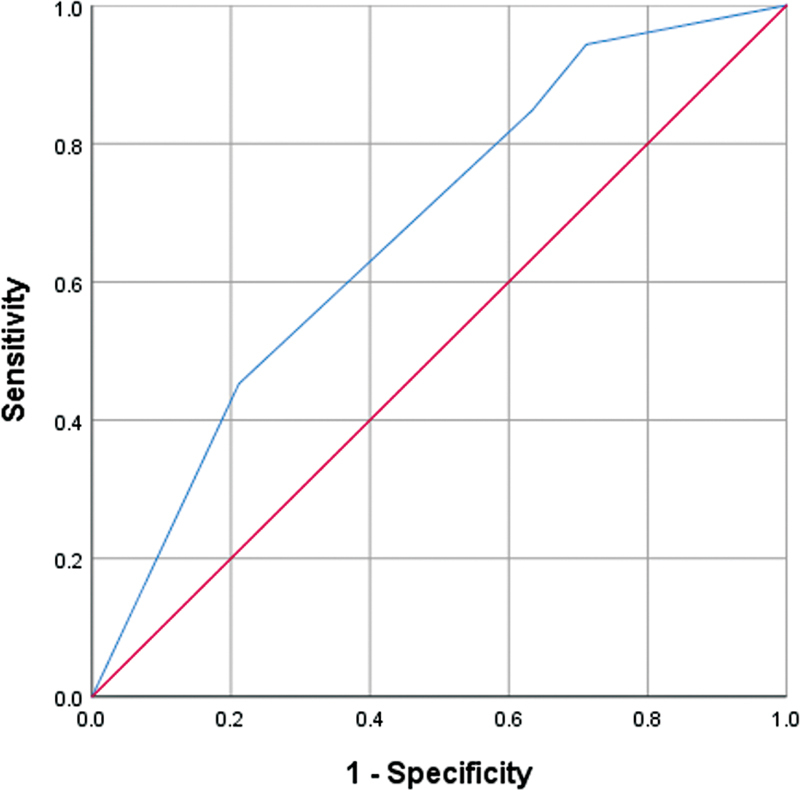
The ROC curves of the multivariate logistic regression. The AUC was 0.673. AUC, area under the curve; ROC, receiver operating characteristic.

Subsequently, multivariate logistic regression was conducted to assess the independent effects of these three variables while controlling for potential confounding factors. The multivariate logistic regression analysis identified two factors that were included in the regression model: a history of chemotherapy and a dosage of hydromorphone ≥ 0.02 mg/kg. The results showed that a history of chemotherapy was associated with a decreased OR (OR 0.325, 95% CI 0.123–0.856, *p* = 0.023), indicating a lower likelihood of the outcome occurring. On the other hand, a dosage of hydromorphone ≥ 0.02 mg/kg was associated with an increased OR (OR 2.857, 95% CI 1.247–6.550, *p* = 0.013), suggesting a higher likelihood of the outcome. The performance of the multivariate logistic regression was evaluated by analyzing ROC curves, resulting in an AUC value of 0.673 ( Fig. 1 ).

## Discussion

This study showed that the incidence of PONV was approximately 50% in female patients undergoing laparoscopic gastrointestinal surgery, even though total intravenous anesthesia and double prophylactic therapy had been administered. A history of chemotherapy was associated with a decreased risk of PONV, and a dosage of hydromorphone ≥ 0.02 mg/kg was associated with an increased risk of PONV.

PONV was a major problem in the perioperative settings, and the incidence of PONV seemed to be extremely high in this trial. The inclusion criteria of this study were female patients undergoing laparoscopic gastrointestinal surgery, with a postoperative analgesic pump containing hydromorphone, and most Chinese females do not have the habit of smoking. All of these factors contributed to the high incidence of PONV. Moreover, a previous study showed that the incidence of PONV was as high as 40 to 53% in the DREAMS trial, which was a large randomized trial that sought to determine whether adding dexamethasone to standard treatment reduced PONV in patients undergoing elective bowel surgery. 5 These results suggested that double prophylactic therapy may not be sufficient to prevent PONV. Further study is needed to determine a multimodal strategy to control PONV better, such as combinations including neurokinin-1 receptor antagonists or olanzapine. 18

Chemotherapy is now widely used before surgery as an effective treatment for many malignancies, but it is associated with significant impacts on organ systems that affect the effect of anesthetics. Wu et al found that chemotherapy in patients with breast cancer could enhance the sedative effect of propofol and shorten the onset time during the induction of anesthesia. 19 As propofol is predominantly metabolized in the liver, the authors suggested that chemotherapy-induced liver damage and nervous system injury may contribute to the enhanced effect of propofol. Propofol has been shown to possess dose-related antiemetic activity, which may thus also be enhanced in patients with a history of chemotherapy. 20 21 The mechanism underlying PONV involves vagal afferents from the gastrointestinal tract and efferent fibers via the vagus nerve and cranial nerves. 22 Chemotherapy can induce both peripheral and central neurotoxicity. 23 24 25 The damage to nerves caused by chemotherapy might also decrease the occurrence of PONV.

Opioids still play a major role in treating postoperative pain, despite opioid-related adverse effects. Thus, doctors must balance the use of opioids to provide sufficient pain relief, while avoiding opioid-related adverse effects. Previous studies have shown a dose–response relationship between postoperative opioid dose and PONV. 26 27 28 However, the exact dose–response relationship between hydromorphone consumption and PONV has not been explored to date. This study offered data on this relationship and showed that a dosage of hydromorphone more than 0.02 mg/kg was associated with an increased risk of PONV, which has not been reported previously.

In our study, the AUC value of our model was 0.673, which can be considered a relatively moderate value. It is worth noting that previous studies have also reported AUC values for various machine learning approaches and scoring systems to predict PONV, ranging from 0.561 to 0.686. 28 29 These values suggest that achieving an ideal AUC for PONV prediction has been challenging. There are multiple factors involved, and the interactions between anesthesia, surgery, and individual characteristics contribute to the complexity. 9 While our study's AUC value may not be ideal, it contributes to the existing body of knowledge on PONV prediction. Further research and exploration in this area are necessary to develop more accurate and reliable models for predicting PONV.

Indeed, this study had certain limitations that should be acknowledged. First, it was a retrospective study, which may introduce selection bias, as the data may not be representative of the entire population. Second, the study was conducted at a single center, which could potentially limit the generalizability of the results. Third, it was worth noting that the sample size in this study was relatively small. Future studies with larger sample sizes and diverse populations are needed to help overcome these limitations and strengthen the evidence base.

## Conclusion

The incidence of PONV remains high in female patients undergoing laparoscopic gastrointestinal surgery, even though total intravenous anesthesia and double prophylactic therapy were administered. A history of chemotherapy and hydromorphone dosage was associated with PONV.
